# A Novel Method for Evaluating Early Tumor Response Based on Daily CBCT Images for Lung SBRT

**DOI:** 10.3390/cancers16010020

**Published:** 2023-12-19

**Authors:** Wei Luo, Zijian Xiu, Xiaoqin Wang, Ronald McGarry, Joshua Allen

**Affiliations:** 1 Department of Radiation Medicine, University of Kentucky, 800 Rose Street, Lexington, KY 40536, USA; zijian.xiu@uky.edu (Z.X.); ronald.mcgarry@uky.edu (R.M.); 2 Department of Radiology, University of Kentucky, 800 Rose Street, Lexington, KY 40536, USA; xiaoqin.wang@uky.edu; 3 AdventHealth, 2501 N Orange Ave, Orlando, FL 32804, USA; joshua.allen@adventhealth.com

**Keywords:** tumor response assessment, stereotactic radiation therapy (SBRT), cone-beam computerized tomography (CBCT), tumor area, tumor linear attenuation coefficient (μ), tumor contrast-to-noise ratio (CNR)

## Abstract

**Simple Summary:**

The assessment of tumor response is important in evaluating cancer treatment and predicting clinical outcomes. The currently used response evaluation criteria in solid tumors (RECIST) are based on tumor size and have limitations in tumor response assessment. We proposed a novel radiologic parameter (R) that combines radiologic tumor changes in size, contrast, and density and utilized patients’ daily cone-beam computerized tomography (CBCT) to evaluate early tumor response to stereotactic radiation therapy (SBRT) for lung cancer. In total, 132 lung cancer patients with 134 tumors were selected for this study. The results of R agreed well with the radiologic assessment performed by an experienced radiologist. Therefore, R can be used for quick, inexpensive, and accurate tumor evaluation for lung SBRT treatment.

**Abstract:**

Background: We aimed to develop a new tumor response assessment method for lung SBRT. Methods: In total, 132 lung cancer patients with 134 tumors who received SBRT treatment with daily CBCT were included in this study. The information about tumor size (area), contrast (contrast-to-noise ratio (CNR)), and density/attenuation (μ) was derived from the CBCT images for the first and the last fractions. The ratios of tumor area, CNR, and μ (R_A_, R_CNR_, R_μ_) between the last and first fractions were calculated for comparison. The product of the three rations was defined as a new parameter (R) for assessment. Tumor response was independently assessed by a radiologist based on a comprehensive analysis of the CBCT images. Results: R ranged from 0.27 to 1.67 with a mean value of 0.95. Based on the radiologic assessment results, a receiver operation characteristic (ROC) curve with the area under the curve (AUC) of 95% was obtained and the optimal cutoff value (R_C_) was determined as 1.1. The results based on R_C_ achieved a 94% accuracy, 94% specificity, and 90% sensitivity. Conclusion: The results show that R was correlated with early tumor response to lung SBRT and that using R for evaluating tumor response to SBRT would be viable and efficient.

## 1. Introduction

The effectiveness of radiation therapy depends on a tumor’s response to treatment. The assessment of tumor response is important in evaluating treatment and predicting clinical outcomes. To minimize observer-related uncertainty in determining tumor response, objective and quantitative criteria should be established and adopted in clinical practice. The currently used objective response evaluation criteria in solid tumors (RECIST) were proposed in 2000 and revised in 2009 [1,2]. These criteria are focused on objective or measurable changes in tumor size. RECIST have related size change (mainly in terms of tumor diameter) to response in the following ways: complete response if tumors disappear, partial response if the tumor diameter in the CT image decreases by 30%, progressive disease if the diameter increases by 20%, and stable disease if the size change is between partial response and progressive disease. While RECIST have been widely used for response assessment, they exhibit uncertainties, such as variability in tumor size measurements and tumoral heterogeneity, which could result in a 30% misclassification rate [3]. Also, the response of tumors to radiation includes not only anatomic changes in tumor size but other biological and radiologic changes as well. Other criteria and methods for tumor response assessment have been introduced to account for the complexity of tumor response, including organ-specific response criteria and functional assessment response criteria [4]. The information used for response assessment can be obtained from pathological study and image analysis. We focused on image analysis in this study.

Various imaging modalities are used for radiation therapy. They include two-dimensional (2D) radiography, three-dimensional (3D) computerized tomography (CT), nuclear magnetic resonance imaging (MRI), and ultrasound. The shapes and sizes of solid tumors are visible and measurable in most of these imaging tools. Other information about biological or physiological changes in tumors may be obtained from MRI and positron-emission tomography (PET) [5,6,7,8]. For example, the F-18 fluorodeoxyglucose (FDG) uptake by tumors in PET contains glucose metabolism [9]. However, radiography, MRI, and PET have many issues, such as significant uncertainties and complexities. Currently, CT is still used as a major imaging tool for the evaluation of tumor response [1,10,11]. However, more information is needed for tumor response evaluation. For example, tumor density (in terms of Hounsfield units (HU) or CT numbers in CT images) was found to be correlated with tumor response [12]. Much more information can be obtained from radiomics, as a large number of radiomic tumor features can be derived from CT images and other images, and some of them may be correlated with tumor response [13,14,15,16,17]. On the other hand, the radiomic features are affected by acquisition modes, reconstruction parameters, smoothing, and segmentation thresholds and can have uncertainties of >30% [13]. Therefore, the accuracy of tumor response assessment should be improved.

Meanwhile, timing should be considered. As tumor response to treatment takes time to develop, a series of CT images should be taken during or after treatment to track tumor changes. Usually, imaging for tumor response assessment is performed at patient follow-ups several weeks, months, and even years after the completion of treatment. Thus, information about very early tumor response may not be available. However, the early detection of tumor change helps with adaptive radiation therapy during treatment and with incorporating additional therapies after treatment to achieve optimal clinical outcomes. The image-guided radiation therapy (IGRT) frequently uses CT and MRI, especially daily kV/MV imaging and cone-beam CT (CBCT), to improve the accuracy of patient positioning and tumor localization. Meanwhile, such images can also be used for tracking tumor change over the entire course of treatment. As a result, very early tumor response during treatment can be observed, and very early tumor response assessment has become possible. 

CT imaging, including CBCT, CT-on-Rails, and Tomotherapy MV CT, is usually performed daily or weekly to ensure accurate patient positioning and tumor localization for intensity-modulated radiation therapy (IMRT), especially in stereotactic radiation therapy (SBRT) treatment. By analyzing the MV CT images on Tomotherapy, Kupelian et al. found that the average decrease in tumor volume was 1.2% per day [18]. Tumor volume change was also observed in CBCT images by Brink et al. [19] and Jabbour et al., who evaluated 38 lung cancer patients treated with 3D conformal radiation therapy [20]. The authors analyzed the weekly CBCT for seven weeks and found a 39.3% decrease in tumor volume between day 1 in the first week and day 43 in the last week, which was consistent with Kupelian’s findings. They further found a correlation between tumor volume decrease and death rate decrease, in that the risk of death decreased by 44.3% for every 10% tumor volume decrease, but no correlation was found between tumor volume and recurrence. Mazzola et al. claimed that 20% of the tumor shrink was correlated with complete response after 6 months [21]. Similar results were obtained by Cremolini et al. [22] and Grewal et al. [23]. Paul et al. also found that a large CT number reduction was correlated with an increase in survival based on CT-on-Rails [24]. Similar results were reported by Wen et al. [25]. Therefore, CBCT is suitable for early response assessment.

In this study, we aimed to introduce a novel tumor response metric and develop a new method that can provide a quick, easy, and accurate determination and prediction of early tumor response to lung SBRT utilizing daily CBCT images within 10 days. 

## 2. Methods and Materials 

### 2.1. Patient Selection

A total of 132 early-stage (stages I and II) non-small-cell lung cancer (NSCLC) patients (ages > 18) with 134 lesions at our institution were randomly selected for this study. The patients were treated with SBRT with 5 fractions (5 × 10 Gy) between April 2018 and June 2022. The patient treatment was performed on Varian TrueBeam every other day. CBCT was taken for patient positioning for each treatment. The CBCT techniques used for lung SBRT included settings of 140 kV, 75 mA, and 1691 mAs. The CBCT images had 512 × 512 pixels and 88 slices with a resolution of 0.9 mm and a slice thickness of 2 mm. The CBCT images for the first and last fractions, as well as the planning CT, were used for analysis in this study. The elapsed time between the first and last fractions was about 8 to 10 days. This is a retrospective study that was approved by our Institutional Review Board (IRB). 

### 2.2. Image Analysis

We selected the CBCT slices that represented the central part of tumors for analysis. Three parameters, tumor target size in terms of area, contrast-to-noise ratio for the target, and Hounsfield unit (HU) of the target, were derived from the CBCT images using ImageJ and the treatment planning system, Varian Eclipse 15.6. The tumor target was identified with reference to the planning CT and was contoured by an iso-pixel line in ImageJ 1.38e for analysis. The area of the tumor target, *A*, was calculated by ImageJ. The mean pixel value, *T*, and the standard deviation, *σ_T_*, were also derived for the contour by ImageJ to represent the target. A region of interest (ROI) in the area surrounding the tumor was defined as the background of the target. The mean pixel value, *B*, and standard deviation, *σ_B_*, calculated for the ROI, represented the background of the target. Then, the contrast-to-noise ratio (CNR) was calculated as follows:

(1) $$CNR=\frac{\left|T-B\right|}{\sqrt{\sigma_{T}^{2}+\sigma_{B}^{2}}}$$

The Hounsfield units (HU) for the target were obtained in Varian Eclipse and converted to a linear attenuation coefficient (*μ*) using the following equation:

(2) $$\mu =(1+\frac{HU}{1000})\;\mu_{water}$$

The highest value of *μ*, which represents the maximum density of the target, was used for analysis in this study. 

### 2.3. Tumor Response Evaluation Parameters

Tumor response during the course of treatment can be measured by the changes in area, contrast, and density between the first treatment and the last treatment, which are described by the ratios of area (A), contrast-to-noise ratio (CNR), and attenuation (μ) between the first fraction (F) and the last fraction (L).

(3) $$R_{A}=\frac{A_{F}}{A_{L}}$$

(4) $$R_{CNR}=\frac{{CNR}_{F}}{{CNR}_{L}}$$

(5) $$R_{\mu }=\frac{\mu_{F}}{\mu_{L}}$$

Then, a new parameter combining the three ratios, *R*, is defined as the product of $R_{A},\;R_{CNR},\;and\;R_{\mu }$ as follows:

(6) $$R=R_{A}\cdot R_{CNR}\cdot R_{\mu }$$

The effect of radiation on tumors can be binarized as response/stable and progression. A cutoff value, *R_C_*, can be derived individually for *R_A_*, *R_CNR_*, *R_μ_*, and *R* to define response/stable (≤*R_C_*) and progression (>*R_C_*).

### 2.4. Radiologic Assessment of Tumor Response

The radiologic assessment of tumor response was performed by an experienced radiologist with more than 10 years of experience using the planning CT and CBCT images, including all the images of the targeted tumor. Radiologic assessment is a comprehensive study utilizing information about tumor size/volume, tumor density, tumor border, morphological features, and tumor change patterns based on the radiologist’s clinical experience. Moreover, locations of the lung nodules, presence of emphysema, ground glass opacities, eccentricity, compactness, roughness, and nodule marginal patterns may also be considered in radiologic assessment [26,27]. The results of the radiologic assessment were classified as response, stable, and regression. In this study, the classifications of response and stable were considered as diseases under control and were combined as one result, such that the radiologic assessment results were binarized as response/stable (controlled) and progression (uncontrolled). Since radiologic assessment performed by radiologists is clinically accepted and has a direct impact on clinical decisions, the results of the radiologic assessment were considered as true clinical results and were used to verify our calculation results in this study.

### 2.5. Statistical Analysis

The parameter, *R*, was correlated with the binary results of the radiologic assessment based on a cutoff, *R_C_*. Given *R_C_*, the quantity accuracy, sensitivity, specificity, positive predictive value, and negative predictive value can be calculated to evaluate the ability of detection of the tumor response with *R*. If the cutoff *R_C_* varies, the values above 5 quantities vary as well. Using the varying sensitivity and specificity values, a receiver operation characteristic (ROC) curve can be plotted. An optimal value of *R_C_* can be derived from the ROC curve and used to assess tumor response with *R*. 

## 3. Results

### 3.1. R_A_, R_CNR_, R_μ_, and R

**Figure 1:**
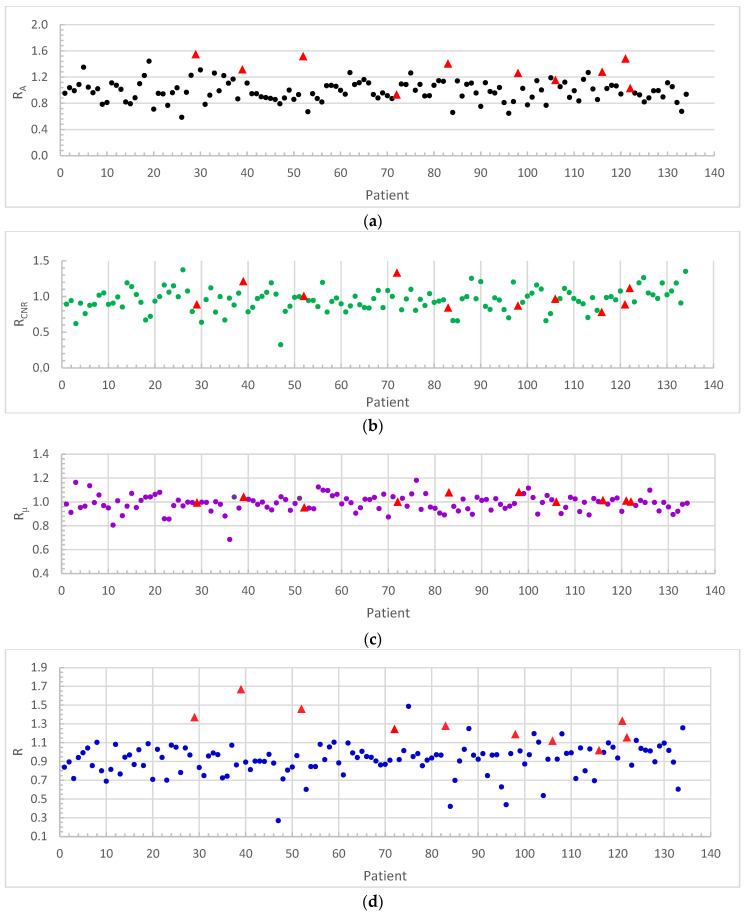
The distribution of (**a**) *R_A_*, (**b**) *R_CNR_*, (**c**) *R_μ_*, and (**d**) *R*. Red triangles represent the progression cases determined by radiologic assessment.

**Figure 2:**
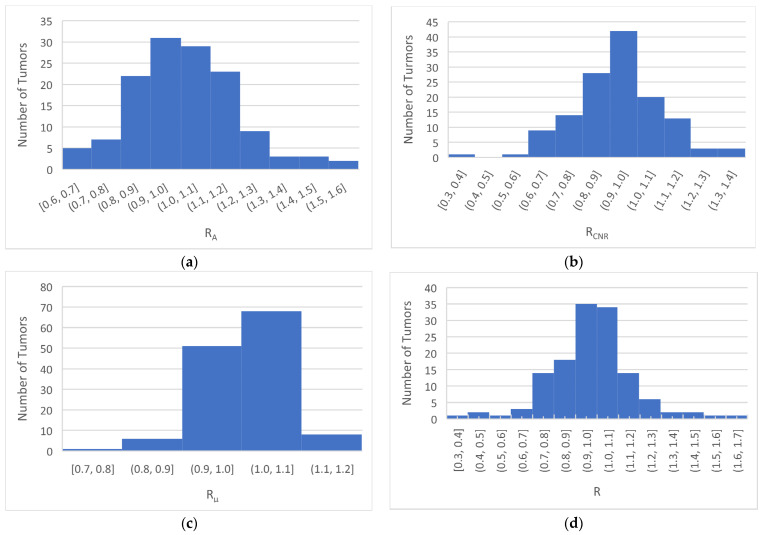
Histograms of *R_A_* (**a**), *R_CNR_* (**b**), *R_μ_* (**c**), and *R* (**d**).

The features of tumors in CBCT images were quantified by the area, CNR, and *μ* of the tumor, typically in a central image slice. The ratios, *R_A_*, *R_CNR_*, *R_μ_*, and *R*, described tumor changes and were derived for each patient in this study. For all 132 patients and 134 tumors included in this study, the values of *R_A_*, *R_CNR_*, and *R_μ_* ranged from 0.59 to 1.55, 0.33 to 1.38, and 0.69 to 1.18, respectively. The mean values were 1.01, 0.96, and 0.99 for *R_A_*, *R_CNR_*, and *R_μ_*, respectively. The values of *R*, the product of *R_S_*, *R_CNR_*, and *R_μ_*, ranged from 0.27 to 1.67 with a mean value of 0.95. The distributions of *R_A_*, *R_CNR_*, *R_μ_*, and R were plotted and are shown in Figure 1a–d. The CBCT images were evaluated by an experienced faculty radiologist, who found 10 progressions, 32 responses, and 92 stables. Progressions are labeled with red triangles in Figure 1. The others were classified as stable/response, and are labeled with blue markers. The corresponding histograms are shown in Figure 2.

### 3.2. The ROC Curves and Cutoffs of Rs

**Figure 3:**
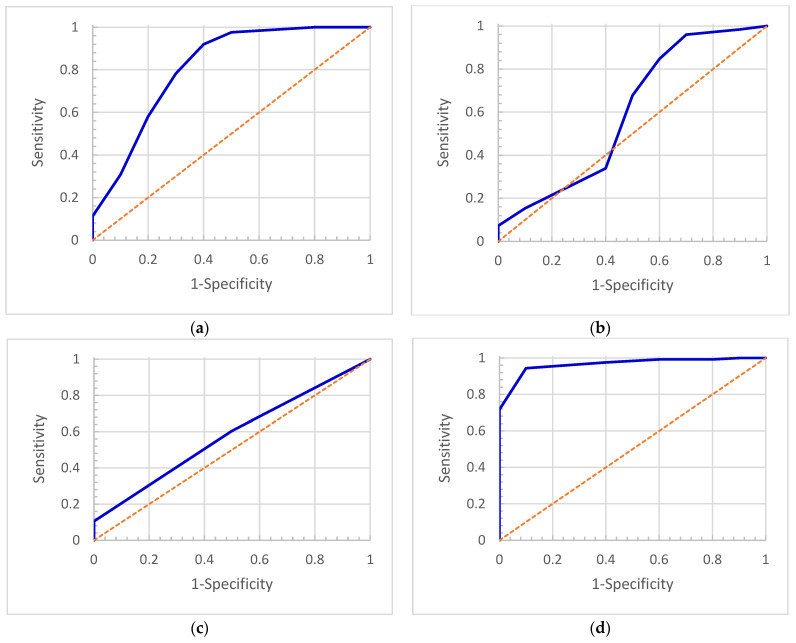
ROC curves for (**a**) *R_A_*, (**b**) *R_CNR_*, (**c**) *R_μ_*, and (**d**) *R*. The dash line is a reference line under which the area is 0.5.

The ROC curves for *R_S_*, *R_CNR_*, and *R_μ_* were obtained by varying the cutoff values of *R_S_*, *R_CNR_*, *R_μ_*, and *R*, respectively. We varied the cutoff values in the entire range of values of *R_S_*, *R_CNR_*, *R_μ_*, and *R* and obtained various values of sensitivity and specificity, which were used to plot the ROC curve, as shown in Figure 3. The optimal cutoffs of *R* were determined as 1.2 for *R_A_*, as 1.0 for *R_CNR_* and *R_μ_*, and as 1.1 for *R*. The areas under the curve (AUC) with a 95% confidence interval (CI) were 0.68 (0.59, 0.75) (*R_A_*), 0.60 (0.51, 0.68) (*R_CNR_*), 0.58 (0.49, 0.66) (*R_μ_*), and 0.95 (0.90, 0.97) (*R*). The optimal cutoff *R_C_* was found for each case based on the analysis of the corresponding ROC curve, as shown in Figure 3. The results of accuracy, sensitivity, specificity, positive predictive value, and negative predictive value corresponding to the optimal cutoff *R_C_* were calculated and are summarized in Table 1, which shows that *R* with *R_c_* of 1.1 gave the best results.

### 3.3. Comparisons

**Figure 4:**
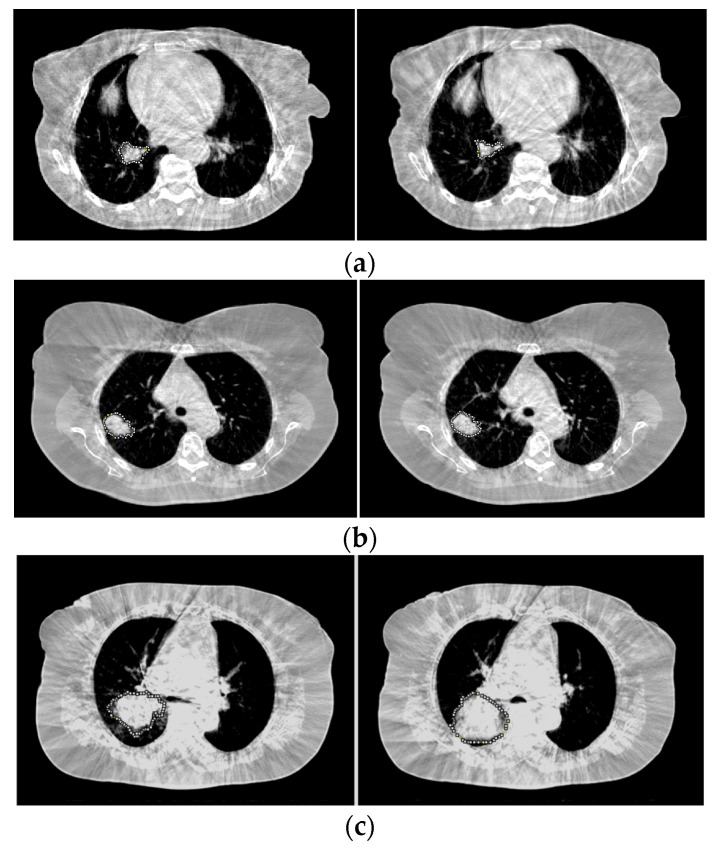
Tumor change between CBCT 1 (**left**) and CBCT 5 (**right**). *R_A_*, *R_CNR_*, *R_μ_*, and *R* are consistent with RA. (**a**) Response; (**b**) stable; (**c**) progression. Tumors are contoured by an iso-pixel line.

**Figure 5:**
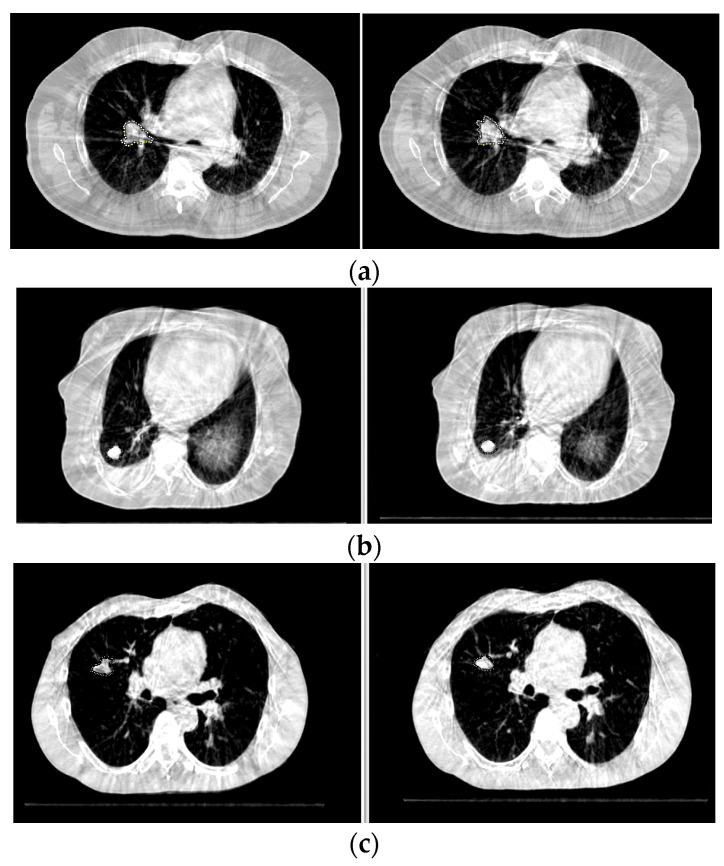
Tumor change between CBCT 1 (**left**) and CBCT 5 (**right**). *R* is consistent with RA, but *R_A_*, *R_CNR_*, and *R_μ_* are not consistent with RA. (**a**) Progression; (**b**) response; (**c**) stable. Tumors are contoured by an iso-pixel line.

Figure 4 shows the CBCT images for three patients where the changes in tumors between the first fraction (CBCT 1) and the last fraction (CBCT 5) are visible. The values of *R_A_*, *R_CNR_*, *R_μ_*, and *R* were consistent with the radiologic assessment (RA) results based on the corresponding *R_C_* values. The results for the three patients are listed in Table 2. Figure 5 displays three other patients and shows that the *R* values with *R_C_* of 1.1 agreed with the RA results, but the results from *R_A_*, *R_CNR_*, and *R_μ_* were different from those of RA. Such results are also included in Table 2.

## 4. Discussion

The assessment of tumor response is complex. While the RECIST are valid in determining tumor response, they have limitations. First of all, they simplify the response evaluation criteria and are limited to tumor size. However, tumor response is associated with other factors, such as tumor density and texture, which are missing in the RECIST. Choi found that assessment based on the RECIST significantly underestimated the early tumor response in patients with advanced gastrointestinal stromal tumor; the study indicated a combination of a 15% or greater decrease in tumor density and a 10% or greater decrease in tumor size on CT was promising when assessing early tumor response and predicting clinical outcomes [28]. Ganeshan et al. found that the CT texture associated with tumor heterogeneity can be used as a quantitative prognostic biomarker for response assessment and survival prediction [29]. Another limitation of the RECIST is that they have a very strict definition of partial response and progression. According to the RECIST, a decrease of only 30% or greater in tumor diameter can be recognized as a partial response, and an increase of at least 20% in tumor diameter can be considered as progression. In a spherical tumor, a decrease of 30% in diameter results in decreases of ~50% in area and ~65% in volume. Similarly, an increase of 20% in diameter is equivalent to increases of over 40% in tumor area and over 70% in tumor volume. If only 20 or 30% or larger changes are recognized as tumor response, a smaller change in tumor size must be ignored by the RECIST. What happens to a patient whose tumor shows changes within 20% to 30%? As a matter of fact, a less than 30% decrease in diameter may still be considered a positive response, and a less than 20% increase in diameter may also lead to progression. Choi found that mean reductions in tumor sizes of approximately 13% were also significant [28]. Moreover, changes over 20 or 30% may not be observed until significant time has passed. Therefore, the RESICT may not be suitable for very early tumor response evaluation. 

Tumor size can also be described by other parameters such as volume. The correlation between tumor volume and clinical outcomes, including survival and risk of death, has been investigated [20,21,22,23,24,25]. Another parameter, HU, which is associated with tumor density, has also been found to be correlated with survival [24,25]. Paul et al. found that HU reduction > 30 HU increased survival by about 13% compared to HU reduction <30 HU [24]. Wen et al. found that combined reductions of 28.44 in HU and 32.01% in volume were associated with response to treatment, while the combined reductions of 19.63 in HU and 23.20% in volume were associated with non-response [25]. However, CT scanners display significant uncertainties. HU variations can be up to 60 HU, and the tolerance of HU uncertainty is determined as 50 HU [30,31]. Thus, the reduction to 30 HU may not be accurately used as a cutoff. 

We have introduced two parameters that are associated with tumor size and tumor density. One is the tumor area ratio, R_A_, and the other is the tumor attenuation ratio, R_μ_. The parameter μ is related to density and can be derived from HU. In our previous study, we found that the change in CNR was correlated with tumor response [32]. We have also introduced the ratio of CNR, R_CNR_. The three parameters each describe tumor change from different aspects and they can be used for tumor response assessment. Based on the analysis of ROCs, the cutoff values for R_A_, R_μ_, and R_CNR_ were 1.2, 1.0, and 1.0, respectively. These values indicate the tumor is in progression if the area of a tumor increases by 20% or if attenuation or CNR increase by any amount. Otherwise, the tumor is stable or responding to radiation therapy. However, the results from R_A_, R_μ_, and R_CNR_ individually did not agree well with the RA results. The AUCs for the three parameters were lower than 0.70, as shown in Figure 3 and Table 1. 

The new parameter, R, proposed in this study combines changes (ratios) in tumor size (area), tumor density (HU/μ), and contrast (CNR) as the product of R_A_, R_μ_, and R_CNR_. R is a single parameter but contains tumor changes in size, density, and contrast. The threshold or cutoff of R_C_ = 1.1 indicated a 10% increase would cause tumor progression. Using R to evaluate tumor change, the results agreed with the RA results very well in terms of high accuracy, high sensitivity, high specificity, and high AUC compared to R_A_, R_μ_, and R_CNR_. More specifically, as shown in Figure 4 and Table 2, R agreed with PA in all three cases, while R_A_, R_μ_, and R_CNR_ all failed in (a), R_μ_ failed in (b), and R_CNR_ failed in (c). In particular, in (a), R_A_ (=1.15) indicated a 15% increase in area or an increase in diameter of about 7.5%, and the disease was determined as progression according to R (=1.12) and RA (=Progression), but it was defined as a stable disease by the RECIST and R_A_. As radiosensitivity affects tumor response, the potential variation in radiosensitivity (e.g., hypoxia) between tumors would change the results of tumor response. However, the method of response assessment using R is not affected.

In this study, the tumor target was defined by a contour that was quantitatively determined by the same pixel value (iso-pixel value). Contouring based on the same pixel value would reduce the chance for human error and has the potential to be computerized and automated. Since the parameters R_A_, R_μ_, and R_CNR_ were derived based on the contour, the calculation of those parameters may also be automated using a computer program. The automation of contouring and calculation will significantly improve the efficiency of and reduce the uncertainty in response assessment. 

Finally, since daily CBCT can be used, the assessment of early tumor response within 10 days of treatment has become available. Specifically, it is quick, convenient, and easy to perform by utilizing routine CBCT images without adding any additional procedures and costs. 

Nonetheless, this study has limitations. First of all, while the parameter R contains tumor size, density, and contrast and was able to provide an accurate assessment of tumor response, studies have shown other factors, such as tumor textures, to be related to tumor response, which are not included in R. In particular, radiomics has been introduced for tumor response and clinical outcome study. Radiomics can derive a large number of features, including tumor textures from CT images or other types of images, which may be correlated with tumor response [13,14,15,16,17]. While radiomics has issues with high uncertainty and low predictability (e.g., most response studies using radiomics resulted in <0.80 in terms of AUC values [15]), radiomics will be considered in our future studies, and some features discovered by radiomics may also be integrated with R. Secondly, the results of tumor change determined in this study were only based on a single radiologist’s clinical experience and judgment. The assessment may include uncertainty, as it is more or less subjective. More tests by multiple observers and multiple institutions would help reduce such uncertainty. Lastly, CBCT artifacts, organ motions, etc., can cause uncertainties, and the tumor geometry and density would likely vary with the patient’s respiration. Firstly, we chose the CT slices with minimum artifacts for analysis. Secondly, this study was based on the comparison of tumor radiologic changes between the first and last fractions. As the CBCT used the same techniques and was performed under the same patient setup conditions for both first and last fractions, certain changes caused by image quality may have counteracted each other. Also, the CBCT images were acquired over ~23 s or 2–3 breathing cycles; it was not a single snapshot that dramatically changed at different times in a breathing cycle but instead included all the variations. Thus, the effect of respiration on the changes in tumor size and density between the first and last fractions in CBCT images was minimized and was ignored in this study.

It should be noted that early tumor changes are not equal to long-term clinical outcomes, but they are strongly correlated. Such correlation can be derived from clinical data analysis [20,21,22,23,24,25] and numerical or dynamic models [33,34]. The correlation between early tumor changes and long-term clinical outcomes can be used for clinical outcome prediction and helps physicians to respond quickly if treatment plans should be adjusted or additional therapies are needed. The correlation between R and long-term clinical outcomes will be further investigated in our future study. 

## 5. Conclusions

Tumor response assessment is complex. The widely used RECIST for tumor response assessment has limitations, and new assessment methods are needed. In this study, we proposed a new method using a novel parameter R that combines tumor size change, tumor density change, and tumor contrast change based on daily CBCT images. The results show that R with a threshold R_C_ was a good metric used to evaluate early tumor response during lung SBRT treatment with high accuracy. The correlation between R and long-term clinical outcomes will be further investigated in our future studies. 

## Figures and Tables

**Table 1:** Statistical results for *R_S_*, *R_CNR_*, *R_μ_*, and *R*.

	*R_A_*	*R_CNR_*	*R* *_μ_*	*R*
*R_C_*	1.2	1.0	1.0	1.1
AUC	0.68	0.60	0.58	0.95
Accuracy	0.90	0.66	0.60	0.94
Sensitivity	0.92	0.68	0.61	0.94
Specificity	0.60	0.50	0.50	0.90
Positive Predictive Value	0.97	0.94	0.94	0.99
Negative Predictive Value	0.38	0.11	0.09	0.56

**Table 2:** Comparison of tumor responses between calculation and radiologic assessment (RA) for R_A_, R_CNR_, R_μ_, and R.

	Patient	*R_A_*	*R_CNR_*	*R_μ_*	*R*	RA
*R_C_*		1.2	1.0	1.0	1.1	
Figure 3	(a)	0.81	0.82	0.88	0.59	R
	(b)	1.04	0.95	0.97	0.96	S
	(c)	1.32	1.21	1.04	1.67	P
Figure 4	(a)	1.15	0.97	1.00	1.12	P
	(b)	0.82	1.19	0.97	0.95	R
	(c)	0.88	0.86	1.13	0.85	S

## Data Availability

The data presented in this study are available in this article.
